# Correction: Identification and functional characterization of key biomarkers in diffuse large B-cell lymphoma: emphasis on STYX as a prognostic marker and therapeutic target

**DOI:** 10.1186/s41065-025-00433-4

**Published:** 2025-04-25

**Authors:** Junaid Abid, Mahmood Basil A. Al-Rawi, Ahmad Mahmood, An Li, Tiemin Jiang

**Affiliations:** 1 State Key Laboratory of Food Nutrition and Safety, College of Biotechnology, Tianjin University of Science and Technology, Tianjin, 300222 China; 2 Department of Optometry, College of Applied Medical Sciences, King Saud University, Riyadh, 11433 Saudi Arabia; 3 Department of Hepatobiliary & Hydatid Diseases, Digestive & Vascular Surgery Center, The First Affiliated Hospital of Xinjiang Medical University, Urumqi, 830054 Xinjiang China; 4 State Key Laboratory of Pathogenesis, Prevention and Treatment of High Incidence Diseases in Central Asia, Xinjiang Medical University Urumqi, Urumqi, 830054 China

**Correction: Hereditas 162, 45 (2025)**

**https://doi.org/10.1186/s41065-025-00411-w**

Following publication of the original article [1], the author reported the following errors.

**Table:** 

Part of the Article	From	To
Authors name	Basil A. Mahmood Al-Rawi	Mahmood Basil A. Al-Rawi
Acknowledgements	None to declare.	The authors of this study extend their appreciation to Researchers Supporting Project (Project number RSP2025R378), King Saud University, Riyadh, Saudi Arabia.
Author’s Email	zhangruiqing0991@163.com	lian861105620@gmail.com
Funding	The authors of this study extend their appreciation to Researchers Supporting Project (Project number RSP2025R378), King Saud University, Riyadh, Saudi Arabia.	This study was supported by the Researchers Supporting Project, King Saud University, Riyadh, Saudi Arabia, (RSP2025R378) which provided funding for this work.

The original article has been updated.
